# The Legal Duties of Hospital Governors

**Published:** 1913-01-25

**Authors:** 


### LEGAL NOTES FOR INSTITUTIONAL WORKERS.

**(The Editor will be pleased to answer Legal queries in this column.)**

### The Legal Duties of Hospital Governors.

In an action for recovery of damages against the directors of the Greenock Hospital the pursuer (plaintiff) averred that, having met with an accident by which the lower part of her thigh bone was fractured, she called in a doctor, who advised her to go at once to the hospital in order to have the advantage of the medical appliances there, and in order to have the injury examined and treated; that she accordingly applied to the hospital, and was received as a paying patient therein at the rate of £2 2s. per week for board and medical treatment by arrangement made with the house surgeon; and that the treatment of her case by the defenders' surgeons was negligent and unskilful, and resulted in injury and damage to her. The general law as to the duty of hospital authorities to their patients was very clearly put in Hillyer *v.* The Governors of St. Bartholomew's, 1909, 2 K.B. 820. They do not undertake the duties of medical men, or to give medical advice, but they do undertake that the patients in their hospitals shall have competent medical advice and assistance. That is to say, in the absence of special contract, the legal responsibilities impliedly attaching to the governors of an infirmary are that they carefully select a competent staff, but that does not amount to a guarantee that the professional actings of the staff will be performed with any specific degree of care and skill in any particular case. The institution offers and undertakes to furnish to the public the services of competent medical and surgical practitioners, and nothing more.

The Rights of Paying Patients.

In the Greenock case, which is reported under the name Foote *v.* Shaw Stewart and Others, 49 S.L.R. 41, an effort was made to distinguish the circumstances there from the St. Bartholomew's case in respect that the infirmary authorities, by receiving a fee of £2 2s. from Mrs. Foote, the pursuer, undertook that the treatment which she should receive from their doctors would be reasonably skilful and careful. Otherwise stated, this argument implied that though to the ordinary non-paying patient the duty of the infirmary trustees was simply to provide the services of a competent staff of medical men, yet to a paying patient they were bound to ensure a special degree of medical care and attention, or a guarantee of skill on the part of the house surgeons. The Court held that, in the absence of a contract much more specific and definite than any that could be implied from the pursuer's averments, they could make no distinction between the case and that of Hillyer *v.* St. Bartholomew's, and they accordingly dismissed it.

It will at once be seen that the Court were fully alive to the importance of the principle of Hillyer's case. Indeed, as one of the judges put it, considerations of reason and good sense seem to negative any further degree of responsibility on the part of the governing body of an infirmary. It is obvious that they have, and can have, no means and no ability of control, no power or capacity of supervision over the professional treatment of their patients. The medical staff must *ex necessitate rei* act on their own responsibility; they are not appointed as in room and stead of the governors to perform duties which the latter might or could themselves undertake. It follows that though the governors appoint and may dismiss the medical staff, they cannot be held responsible for the degree of skill or care exercised by the latter in the treatment of any patient. They undertake to furnish their patients with the services of a competent staff; they do not undertake indemnity against errors of treatment arising from want of skill or negligence on the part of practitioners whom they have reasonably and properly appointed as persons competent for their posts.

The Liability of Assistant Surgeons.

It was probably the full and emphatic statement of law contained in the two decisions, on which we have just commented, that caused the action Frame *v.* McLennan, 1912, 2 S.L.T. 359, in which an infirmary patient alleged that he had sustained injury and damage in consequence of an operation unskilfully performed by one of the hospital surgeon's assistants, to be laid not against the infirmary but against the surgeon. The action was a Sheriff (County) Court one. In dismissing the case Sheriff Fyfe made the following analysis of the pursuer's pleadings:—First, the proposition of the pursuer seemed to be that each of the consulting surgeons in a large charitable institution, like the Western Infirmary, Glasgow, accepts personal liability for all the work done by any of the assistant surgeons. "That," remarked the Judge, "seems to me a perfectly preposterous suggestion." Second, there was the question of the right and title of the pursuer to raise such an action; and on that point the Court was of opinion that a patient who is accorded the privilege of treatment at an infirmary has no title to sue such an action, there being no contract between him and the individual infirmary surgeon.

